# Prevention and Management of Peptide Receptor Radionuclide Therapy-Induced Hypertensive Crisis in a Patient With Metastatic Pheochromocytoma

**DOI:** 10.1016/j.aed.2025.11.001

**Published:** 2025-11-13

**Authors:** Run Yu, Linda Gardner, Ali Salavati, Shadfar Bahri

**Affiliations:** 1 Division of Endocrinology, UCLA David Geffen School of Medicine, Los Angeles, California; 2 Ahmanson Translational Imaging Division, UCLA David Geffen School of Medicine, Los Angeles, California

**Keywords:** hypertensive crisis, lutetium-177 DOTATATE, metastatic pheochromocytoma, metyrosine, peptide receptor radionuclide therapy

## Abstract

**Background/Objective:**

While peptide receptor radionuclide therapy (PRRT) with lutetium-177 DOTATATE has been increasingly used for metastatic pheochromocytoma management, PRRT-induced hypertensive crisis has been documented in prior reports. A case is reported here to demonstrate the prevention and management of PRRT-induced hypertensive crisis.

**Case Report:**

A 75-year-old male developed PRRT-induced hypertensive crisis. He had known progressive metastatic pheochromocytoma. Despite scheduled daily alpha and beta blockade, he developed symptomatic hypertensive crisis shortly after completion of lutetium-177 DOTATATE infusion in the first 2 treatments, which was managed with as-needed oral alpha blockade. Additional alpha blocker was given right before the initiation of lutetium-177 DOTATATE infusion in the third and fourth treatments. Hypertensive crisis still occurred in the third but not in the fourth treatment. He required the fifth treatment due to tumor progression but he developed another hypertensive crisis despite additional alpha blockade right before treatment. The patient became very concerned for potential hypertensive crisis in the sixth treatment. He was treated with escalating dose of metyrosine for 4 days before the treatment and did not develop hypertensive crisis. No additional alpha blockade was given.

**Discussion:**

PRRT may directly stimulate catecholamine release or cause tumor lysis in patients with metastatic pheochromocytoma, resulting in hypertensive crisis.

**Conclusion:**

PRRT-induced hypertensive crisis can happen in patients treated for metastatic pheochromocytoma. If hypertensive crisis occurs, caution should be taken on the subsequent treatments. Additional alpha blockade right before lutetium-177 DOTATATE infusion may help; in challenging cases, short-term metyrosine use may prevent PRRT-induced hypertensive crisis.

**Highlights**

• Peptide receptor radionuclide therapy (PRRT)-induced hypertensive crisis is a rare but severe complication in patients with metastatic pheochromocytoma
• Ample pre-PRRT alpha and beta blockade are essential to prevent hypertensive crisis
• In challenging cases, metyrosine can be used in short term to prevent hypertensive crisis
• Hypertensive crisis is managed by additional alpha and beta blockade

**Clinical Relevance**

Peptide receptor radionuclide therapy (PRRT)-induced hypertensive crisis can happen in patients treated for metastatic pheochromocytoma. If hypertensive crisis occurs, caution should be taken on the subsequent treatments. Additional alpha blockade right before lutetium-177 DOTATATE infusion may help; in challenging cases, short-term metyrosine use may prevent PRRT-induced hypertensive crisis.

## Introduction

Hypertensive crisis in patients with pheochromocytoma can happen during anesthesia, endoscopy, and surgical procedures, due to acute release of catecholamines from pheochromocytoma triggered by the procedures.^1^^,^^2^ Hypertensive crisis can also happen in patients with metastatic pheochromocytoma.^3^ Peptide receptor radionuclide therapy (PRRT) with lutetium-177 DOTATATE has been increasingly used for metastatic pheochromocytoma management.^3^^,^^4^ Hypertensive crisis associated with this therapy is also recognized.^5^, ^6^, ^7^, ^8^, ^9^ Although generally found to be rare, hypertensive crisis can be frightening to patients and nuclear medicine staff, and occasionally lead to severe consequences. There has not been a standard protocol to prevent or manage hypertensive crisis in patients treated with PRRT for metastatic pheochromocytoma. Here we describe our experience in a case report.

## Case Report

**Fig. 1:**
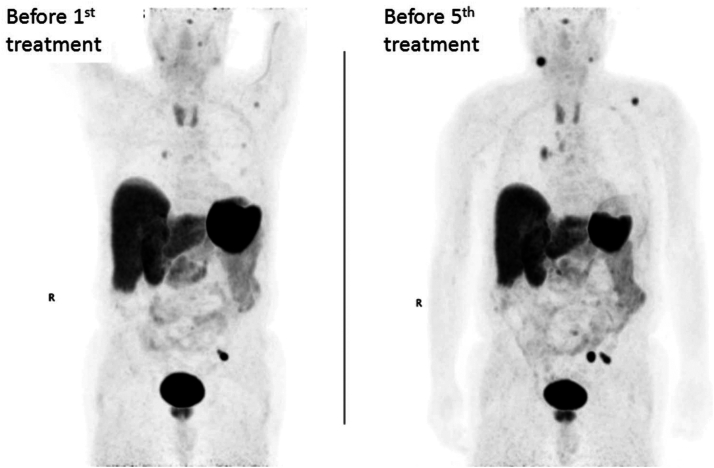
DOTATATE PET before the first and the fifth lutetium-177 DOTATATE treatments. Shown are maximal intensity projection images. *Left* = 5 months before the first treatment; *Right* = 1 month before the fifth treatment.

A 75-year-old male presented with hypertensive crisis during PRRT with lutetium-177 DOTATATE. He had been diagnosed with left adrenal pheochromocytoma and undergone left adrenalectomy at age 55. At age 69, symptoms reemerged and pheochromocytoma recurrence was found in left adrenal bed with metastasis in lymph nodes and multiple bony sites, for which he received surgical and radiation therapies. At age 73, 2 years before presentation, blood pressure and pulse were well controlled by prazosin 2 mg twice daily and metoprolol 25 mg daily; metanephrine was <25 pg/mL (normal <57) and normetanephrine 457 pg/mL (normal <148). As his overall tumor burden then was small, he was monitored for tumor progression. Five months before presentation, metanephrine was 55 pg/mL and normetanephrine 1202 pg/mL. DOTATATE PET showed increased tumor burden (Fig. 1, *Left*). One month before presentation, normetanephrine rose to 1329 pg/mL (Table 1). Metoprolol dose was increased to 50 mg twice daily. He did not have family history of pheochromocytoma. He harbored a T234A likely pathologic variant of *FH*, a known pheochromocytoma-predisposition gene.

**Table 1:** Blood Pressure Management Before and During Lutetium-177 DOTATATE Treatments

Timeline	Pre-treatment metanephrines	Scheduled daily alpha and beta blockade	Pre-treatment with metyrosine	Additional alpha blockade during treatment
1^st^ treatment (199.8 mCi over 60 min)	MN 40 pg/ml, NMN 1329 pg/mL, 1 mo before treatment	Prazosin 2 mg bid, metoprolol 50 mg bid	None	Prazosin 4 mg 45 min after completion of infusion
2^nd^ treatment (199.4 mCi over 71 min, 2 mo after the 1st)	MN 40 pg/mL, NMN 1329 pg/mL, 3 mo before treatment	Prazosin 1/2 mg bid, metoprolol 50 mg bid	None	Prazosin 2 mg 15 min and 40 min after completion of infusion
3^rd^ treatment (199.9 mCi over 57 min, 4 mo after the 1st)	MN 70 pg/mL, NMN 1742 pg/mL, 6 d before treatment	Prazosin 1/2 mg bid, metoprolol 50 mg bid	None	Prazosin 4 mg right before initiation of infusion
4^th^ treatment (196.5 mCi over 60 min, 6 mo after the 1st)	MN 70 pg/mL, NMN 1742 pg/mL, 2 mo before treatment	Prazosin 1/2 mg bid, metoprolol 50 mg bid	None	Prazosin 4 mg right before initiation of infusion
5^th^ treatment (199.8 mCi over 79 min, 21 mo after the 1st)	MN 0 pg/mL, NMN 3322 pg/mL, 2 mo before treatment	Doxazosin 2/1 mg bid, metoprolol 50 mg bid	None	Prazosin 4 mg 1 h before initiation of infusion and 45 min after completion of infusion
6^th^ treatment (199.4 mCi over 55 min, 24 mo after the 1st)	MN 42 pg/mL, NMN 4405 pg/mL, 1.5 mo before treatment	Doxazosin 2 mg bid, Metoprolol 50 mg bid	Yes	None

Abbreviations: MN = metanephrine (normal < 57 pg/mL); NMN = normetanephrine (normal < 148 pg/mL).

**Fig. 2:**
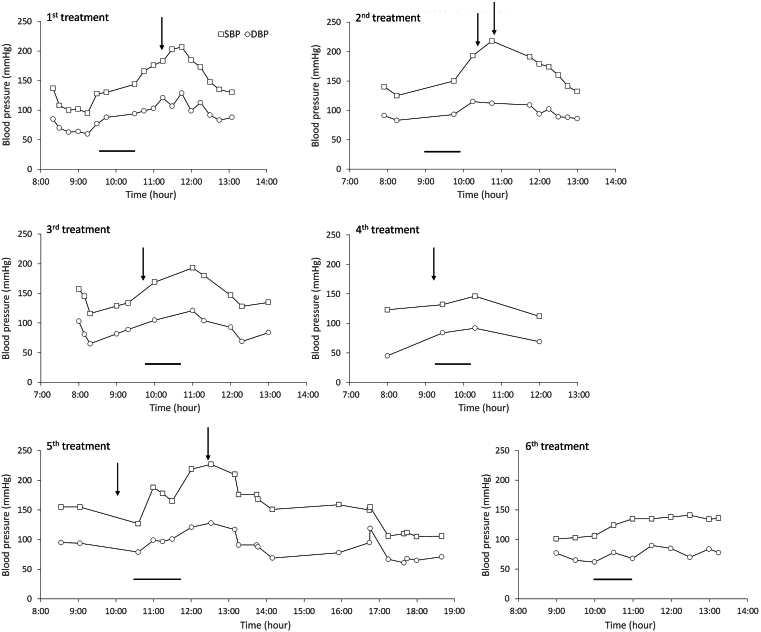
Blood pressure during lutetium-177 DOTATATE treatments. *Bars* indicate lutetium-177 DOTATATE infusion. *Arrows* indicate when prazosin was given. See text and Table 1 for details.

As the patient exhibited a moderate progression of metastatic pheochromocytoma and somatostatin receptor tumor expression was confirmed by DOTATATE PET, PRRT was favored over chemotherapy or targeted therapies.^3^^,^^4^ He thus underwent PRRT. During the first lutetium-177 DOTATATE treatment, his blood pressure began to rise after the completion of lutetium-177 DOTATATE infusion (Fig. 2) and he reported headache and anxiety. His endocrinologist was immediately notified. Prazosin 4 mg was given 45 min after completion of the infusion (Table 1 and Fig. 2). His systolic blood pressure peaked at 207 mmHg before gradually returning to normal range over 2 h, and his symptoms abated as well. He was discharged home in stable conditions. Due to lower blood pressure after the first treatment, prazosin dose was decreased to 1 mg in morning and 2 mg in evening. Two months later, he underwent the second treatment. Again his blood pressure began to rise after the completion of lutetium-177 DOTATATE infusion with peak systolic blood pressure 218 mmHg, and he reported headache and anxiety (Fig. 2). Prazosin 2 mg was given 15 min and 40 min, respectively, after completion of the infusion; his blood pressure gradually returned to normal and symptoms abated. Two months later, his normetanephrine further increased to 1742 pg/mL (Table 1); he underwent the third treatment. In consideration of hypertensive crisis in the 2 previous treatments, Prazosin 4 mg was given right before the initiation of lutetium-177 DOTATATE infusion. Blood pressure still rose during and after completion of the infusion with peak systolic blood pressure 193 mmHg but gradually returned to normal (Fig. 2). Two months later, prazosin 4 mg was given right before the initiation of the fourth lutetium-177 DOTATATE infusion; hypertensive crisis did not happen; the peak systolic blood pressure was 146 mmHg after completion of the infusion (Table 1 and Fig. 2).

His normetanephrine level exhibited no significant change for 6 months: 1721 and 1701 pg/mL at 3 and 6 months after the fourth treatment, respectively. Three months later (9 months after the fourth lutetium-177 DOTATATE treatment), normetaneprine rose to 2053 pg/mL. DOTATATE PET showed grossly stable disease. Four months later (13 months after the fourth treatment), normetaneprine further rose to 3322 pg/mL. DOTATATE PET showed increased disease burden (Fig. 1, *Right*). Blood pressure and pulse were well controlled by doxazosin 2 mg in morning and 1 mg in evening (the switch from prazosin to doxazosin was required by his medical insurance) and metoprolol 50 mg twice daily. Although doxazosin, with its longer half-life, can be taken once daily, the patient preferred taking doxazosin twice daily as he often experienced blood pressure fluctuations and wished to adjust doxazosin dose quickly. He underwent the fifth treatment as salvage therapy. Unfortunately, extravasation of lutetium-177 DOTATATE occurred during the infusion. Despite prazosin 4 mg given right before the initiation of infusion and 45 min after completion of infusion, he developed a hypertensive crisis with peak systolic blood pressure 227 mmHg. He was eventually discharged home in stable conditions. The patient became very concerned for potential hypertensive crisis in the sixth treatment. Doxazosin dose was raised to 2 mg twice daily. Metyrosine was ordered. The patient had to get special insurance approval and pay thousands of dollars out of pocket for metyrosine. He took an escalating dose of metyrosine: 250 mg every 6 hours on day 4 before treatment, 500 mg every 6 hours on day 3, 750 mg every 6 hours on day 2, 1000 mg every 6 hours on day 1, and 1000 mg on the morning of the day of the sixth treatment. He had severe fatigue after taking metyrosine but otherwise tolerated it. Hypertensive crisis did not happen after completion of the infusion (peak systolic blood pressure 141 mmHg). No additional alpha blockade was given. The patient was pleased that hypertensive crisis did not happen.

## Discussion

In this case report, we describe our experience on the prevention and management of hypertensive crisis during PRRT with lutetium-177 DOTATATE for metastatic pheochromocytoma. As hypertensive crisis can happen during anesthesia, endoscopy, and surgical procedures,^1^^,^^2^ whether PRRT would elicit hypertensive crisis has been a concern since its use in metastatic pheochromocytoma.^4^^,^^5^ Early case reports described that hypertensive crisis can happen within hours to days after PRRT agent infusion.^10^^,^^11^ Recent series demonstrated that hypertensive crisis is actually rare in patients with metastatic pheochromocytoma treated with PRRT, and only happens in 3 of 78 patients (3.8%) pooled from 4 series.^6^, ^7^, ^8^, ^9^ The mechanism underlying PRRT-induced hypertensive crisis is not very clear. It is presumed to be acute catecholamines release elicited by PRRT, either through direct stimulation or tumor lysis.^10^^,^^11^ Although PRRT-induced hypertensive crisis tends to occur in patients with biochemically active pheochromocytomas, like in our patient, no other clear patient factors, such as age, sex, tumor size or location, or metanephrines levels, have been found to be predictors of PRRT-induced hypertensive crisis.^6^, ^7^, ^8^, ^9^ Once PRRT-induced hypertensive crisis occurs, it tends to recur with subsequent treatments, as seen in our patient.^6^, ^7^, ^8^, ^9^, ^10^, ^11^

Although rare, PRRT-induced hypertensive crisis is a serious complication. In extreme cases, PRRT-induced hypertensive crisis can be even fatal.^12^ It is generally recommended that patients should be amply treated with alpha and beta blockade before undergoing PRRT.^4^^,^^5^ Slower lutetium-177 DOTATATE infusion over 1 to 2 hours is also recommended. Our patient was treated with both alpha and beta blockade before PRRT and his blood pressure and pulse were both within goals, and our standard infusion duration is over 1 hour. Despite these, our patient still developed hypertensive crisis after the infusion. Additional alpha blockade right before lutetium-177 DOTATATE infusion was used in the third and fourth treatments with variable success. Metyrosine is an inhibitor of tyrosine hydroxylase, the rate limiting enzyme in catecholamines synthesis; it reduces catecholamines synthesis by 80% after short-term use.^13^^,^^14^ Metyrosine is indicated for challenging preoperative preparation and control of severe symptoms in patients with metastatic pheochromocytoma.^13^^,^^14^ Metyrosine, however, is very expensive and not regularly available, and has significant side effects such as severe fatigue. To our knowledge, metyrosine has not been reported as a means of preventing PRRT-induced hypertensive crisis before this case. As our patient became very anxious after repeated hypertensive crises, he eventually was treated with metyrosine for 4 days immediately before the sixth treatment. Although metyrosine was costly for the patient and caused severe fatigue, it indeed appeared to prevent PRRT-induced hypertensive crisis in this patient. PRRT-induced hypertensive management depends on the overall clinical scenario.^4^^,^^5^ Additional oral alpha and beta blockade can be used in patients who are in guarded conditions but expected to recover in hours. Patients with very severe symptoms and who do not respond to oral alpha and beta blockade should be admitted and receive intravenous alpha and beta blockade or vasodilators.

## Conclusion

This case highlights that, although rare, hypertensive crisis induced by PRRT with lutetium-177 DOTATATE can happen in patients treated for metastatic pheochromocytoma (including paraganglioma). Patients who will undergo PRRT should be amply treated with alpha and beta blockade before PRRT and be closely monitored for hemodynamics and symptoms during PRRT. Additional alpha blockade right before lutetium-177 DOTATATE infusion may help; in challenging cases like in our patient, short-term metyrosine use may prevent PRRT-induced hypertensive crisis.

## Funding Statement

This research did not receive any specific grant from funding agencies in the public, commercial, or not-for-profit sectors.

## Patient Consent

The authors acknowledge that patient consent was obtained.

## Conflict of Interest Statement

The authors have no conflict of interest to disclose.
